# Designing and Analyzing Clinical Trials with Composite Outcomes: Consideration of Possible Treatment Differences between the Individual Outcomes

**DOI:** 10.1371/journal.pone.0034785

**Published:** 2012-04-17

**Authors:** Janice Pogue, P. J. Devereaux, Lehana Thabane, Salim Yusuf

**Affiliations:** 1 Department of Clinical Epidemiology and Biostatistics, McMaster University, Hamilton, Ontario, Canada; 2 Faculty of Health Sciences, McMaster University, Hamilton, Ontario, Canada; Copenhagen University Hospital Gentofte, Denmark

## Abstract

When the individual outcomes within a composite outcome appear to have different treatment effects, either in magnitude or direction, researchers may question the validity or appropriateness of using this composite outcome as a basis for measuring overall treatment effect in a randomized controlled trial. The question remains as to how to distinguish random variation in estimated treatment effects from important heterogeneity within a composite outcome. This paper suggests there may be some utility in directly testing the assumption of homogeneity of treatment effect across the individual outcomes within a composite outcome. We describe a treatment heterogeneity test for composite outcomes based on a class of models used for the analysis of correlated data arising from the measurement of multiple outcomes for the same individuals. Such a test may be useful in planning a trial with a primary composite outcome and at trial end with final analysis and presentation. We demonstrate how to determine the statistical power to detect composite outcome treatment heterogeneity using the POISE Trial data. Then we describe how this test may be incorporated into a presentation of trial results with composite outcomes. We conclude that it may be informative for trialists to assess the consistency of treatment effects across the individual outcomes within a composite outcome using a formalized methodology and the suggested test represents one option.

## Introduction

It is common to use primary composite outcomes in trials designed to test the effectiveness of new therapies in preventing or treating disease. Trialists identify a list of outcomes to include in this composite that are thought to share the same disease pathways and therefore, should show similar treatment effects, at least in direction [1]–[7]. This type of composite outcome then assumes homogeneity of treatment effect for all outcomes included in it. However, this assumption can be challenged at the end of the trial, when there is visible variation in the treatment effects among these individual outcomes. Currently there are few formal methods to determine if such variation is due to chance alone, or represents new, unanticipated information that should change how we interpret the overall treatment effect in a trial. This determination is critical as an unanticipated treatment difference between outcomes may alter our assumptions about mechanisms of action potentially for both the treatment and the disease process itself. Without using a formal statistical method to determine if the outcomes within a composite share a common treatment effect, individual readers may come to different conclusions, based solely on variation in judgment. Given the importance of determining if a primary composite outcome can validly represent the overall treatment effect of an intervention, perhaps a more objective assessment should be used.

It is easier to interpret the treatment effect for a primary outcome where there is little variation in this effect for its individual outcomes. The question remains open as to how consistent these individual treatment effects need to be before we should be concerned about using a primary composite outcome to summarize the overall treatment effect. It has been suggested that we may only accept the overall treatment effect if at least one individual outcome also show statistically significant benefit [8]. Another view indicates that all individual outcomes contained in the composite should have point estimates trending in the direction of benefit [9], [10]. Yet we know that power for these individual outcome comparisons will be low in a trial designed to have good power only for the primary composite outcome. Random variation alone can easily result in variation in treatment effects for individual outcomes, as we see so often in under-powered subgroup analyses[11], [12].

Ferreira-Ganzalez et al.[2] suggest using a gradient of efficacy across the individual outcomes for a composite. This gradient is defined as the difference between smallest and largest individual outcome treatment effects, and these differences are then organized into small, moderate or large categories. The limitation of this method is that it does not take variability or the amount of information into account, so can not distinguish random variation from systematic differences.

We suggest that a composite outcome treatment heterogeneity test can be used to clarify variation in treatment effect for the individual outcomes with that composite. A heterogeneity or interaction test is routinely used in both meta-analysis[13] and subgroup[11], [12] analysis to distinguish random variation from systematic differences, and this determination is wisely based on the amount of information available in the analysis. However, with multiple outcomes recorded for the same trial participants, one cannot merely use a simple Cochran's Q test to detect differences in treatment effect across outcomes. The individual outcomes within a composite are correlated with one another and we need to use statistical models that account for this correlation. The purpose of this paper is to illustrate the use of appropriate statistical methods to assess treatment heterogeneity in both the design and analysis of a trial that uses a composite outcome.

Sometimes composite outcomes are formed to quantify risk-benefit or capture competing risks. In these cases, there is no expectation that the treatment will have the same effect on each outcome within the composite. In fact, often it is expected that a new therapy may have greater efficacy and greater harm, than a standard one. In such a case, there is no assumption of homogeneity of treatment effects across the composite components and the methods proposed in this article would not be appropriate.

To illustrate this methodology we use the composite outcome from the POISE Trial [14] as an example. Given our a priori assumption that all components of this composite outcome would share the same direction and approximate magnitude of treatment effect, we present a statistical analysis to address the possible contradiction of this assumption in the design and analysis stages.

## Methods

**Figure 1:**
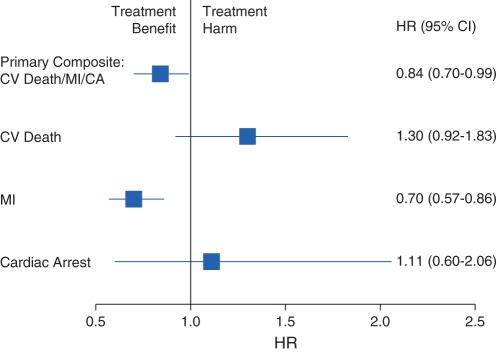
POISE [**14**] results for the primary composite outcome and individual component outcomes. Hazard ratios and 95% confidence interval for time-to-first composite outcome and for each individual outcome within this composite.

The POISE trial [14] examined the effect of peri-operative beta-blocker versus placebo in participants at risk of cardiovascular events who were undergoing non-cardiac surgery. 8351 participants were randomized from 190 centers in 23 countries. The primary composite outcome was time to first occurrence of non-fatal myocardial infarction, non-fatal cardiac arrest, or cardiovascular death within 30 days from randomization. The primary analysis used a Cox regression for the treatment comparison of time to first composite outcome. Results, published previously [14], visually display a lack of homogeneity of treatment effect across the components of the composite outcome (see figure 1).

We would like to fit the following general model:
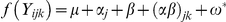
For the ith patient, all outcome types included in the composite outcome are analyzed in a single regression. A function (f) of the outcome for each component of the composite Y_ijk_, is estimated from the following terms: α_j_ represents the treatment effect for j treatment groups, β_k_ is the effect of each individual outcome of the composite outcome for k individual outcome components, (αβ)_jk_ is the interaction of treatment and individual outcomes, intercept μ, and ω* is an error term whose structure will depend on the exact model used. The test of whether the interaction term (αβ)_jk_ is different from zero is the test of homogeneity of treatment effect across the individual components of the composite outcome.

A trial where multiple outcomes are evaluated for the same participants can be viewed as a repeated measures design. These models include terms to account for the non-independence of these data due to an association or correlation of the multiple outcomes (i.e. components of a composite outcome) within a participant. Regardless of the outcome type (binary, continuous, or time to event) there are generally two statistical models used for this type of analysis: random effects and marginal models. For random effects, also known as mixed models, a term for individual variation is incorporated in the model, usually to allow the slope of the regression to vary across participants. Individuals are considered to be randomly selected from a population with an intercept assumed to follow a known distribution [15]. For the current case this model would include a random intercept term γ_i_ assumed to vary for each patient from a common statistical distribution and an error term ε_ijk_:
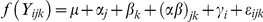



For the marginal or population-averaged model, the association of multiple outcomes within an individual is treated as a nuisance factor and treatment effects are then estimated by averaging over the variability due to the individual, or are obtained at the margin [16]. Thus, the expectation of Y_ijk_ is modeled as follows:
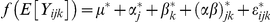



The coefficients from these two models have different interpretations. The marginal model, the * indicates that the coefficients are averaged effects, while the random effects model produces effects specific to the individuals in the analysis.

Statistical models such as these may be used for many different types of composite outcomes. A composite outcome may be formed from a number of continuous outcomes, such as multiple disability scales, that are analyzed as a global test[17]. For continuous and normally-distributed composite outcomes, f() would be the identity link and both marginal and random effects models would be multivariate linear models of the types commonly used for analysis of repeated observation on the same individuals[18]. For the case of a binary composite outcome, f() would be the logit function for a logistic regression. This generalized linear model for binary outcomes analyzes the probability of occurrence of the different outcome types, the effect of treatment, and their interaction on the logit scale. This interaction term would form the composite outcome treatment heterogeneity test. For binary composite outcomes, we have previously demonstrated that a marginal logistic regression model using generalized estimating equations [GEE] [16] had the greatest power to detect composite treatment heterogeneity [19], compared to the random effects model [15], and the weighted logistic regression model, weighted by either the intra-class correlation coefficient [20] or equivalently the variance inflation factor [21]. For time to event data, either the random effects frailty models [22] or marginal models such as that proposed by Wei, Lin and Weissfeld [23], [24] may be used to analyze multiple event time data. Both frailty models and marginal models have been shown to be useful in detecting treatment heterogeneity between the individual outcomes within a composite outcome [25].

Using such a model for repeated or correlated outcome data, we can calculate the power to detect possible heterogeneity of treatment effect across the individual outcomes of the composite outcome at the design stage of a trial. For example, for a time to event composite outcome, we begin with estimated associations between outcome survival times, and then simulate correlated outcome data in order to calculate our chances of detecting a different treatment effect for one individual outcome within the composite outcome. Estimates of the association in survival times for individual outcomes may be taken from existing trials or databases of similar trial participants. Simple correlated time-to-event data may be simulated by creating a Cox proportional hazards model [26] that contains a random frailty term sampled from an assumed distribution (e.g. gamma) to represent the association between two survival times within an individual [22]. However, for greater than two outcomes with different associations between them, simulation of multivariate survival data may be best done through the marginal model. Lin and Wei [23], [24], [27] in developing a marginal model for multivariate time-to-event data, assumed the regression coefficients followed an approximately multivariate normal distribution and then derived a “working” correlation matrix to adjust the covariance matrix estimates for correlated data. The results are known as a “sandwich” estimator or “robust” covariance matrix. Using an estimated robust covariance matrix from a prior dataset and assuming normality of the regression parameters, one can sample from this multivariate normal distribution and insert these within the Cox proportional hazards model [26] to generate random multivariate time-to-event data, provided that the estimated covariance matrix is positive-semi definite [25].

Suppose we were to design a two-group trial in a similar population to the POISE trial [14] with the same composite outcome of first occurrence of non-fatal myocardial infarction, non-fatal cardiac arrest, or cardiovascular death within 30 days from randomization. Assume that during the study, myocardial infarction (MI), cardiac arrest, and cardiovascular death will be experienced by 6%, 0.5%, and 1.5% of the control group participants, respectively. Also we assumed a further 1% of individual will die of a non-cardiovascular cause. From POISE [14] data, we could fit a marginal model to obtain an estimate of the covariance matrix, adjusted for multiple outcomes per participant. For the ith person, kth outcome type, and jth treatment group, this model would include time to event for each of the three outcomes per person (T_1i_, T_2i_, T_3i_) and three classification variables (Y_1i_, Y_2i_, Y_3i_), indicating whether each Tik represents an occurrence of the respective event time or a censoring time due to end of follow-up. Covariates in this regression would include treatment group [Gj = 0 (control) or 1 (active)] and variables that compare the different outcomes to one another [O1 = 0(MI) or 1(cardiovascular death), O2 = 0(MI) or 1(cardiac arrest)]. The following proportional hazards model would be fit:
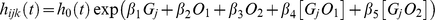



In this model, h_0_(t) represent the risk or hazard of having an MI in the control group. The estimate of β_1_ represents the treatment effect on the MI outcome, while β_2_ and β_3_ represent the difference in risk or hazard between cardiovascular death and MI, and cardiac arrest and MI, respectively. The interaction term β4 estimates the difference in treatment effect between cardiovascular death and MI, and lastly, the interaction term β5 compares the difference in treatment effect between cardiac arrest and MI. A treatment heterogeneity test for the composite outcome would test whether there are any significant differences between the three individual outcomes in their treatment effect (testing the hypothesis that β4 = β5 = 0).

Given a robust estimated covariance matrix Σ and estimates of h_0_(t), β_2_, β_3_ from POISE [14], we can assume a common treatment effect or hazard ratio (λ) for all three outcomes, and set β_1_ = ln(λ), β4 = 0, and β5 = 0. We can then vary the effect on a single interaction term (e.g. β4>0) to see what degree of heterogeneity we may have reasonable power to detect in our future trial. Given these estimates, we assumed that β1, β2, β3, β4, and β5 were multivariate normal with estimated robust covariance Σ (see table 1) and drew random samples of size 8,200 (4100 active and 4100 control participants) from this multivariate distribution to represent simulated participants in our new trial.

**Table 1:** Estimated robust covariance matrix Σ.

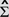 =	σ^2^_β1_	σ_β1β2_	σ_β1β3_	σ_β1β4_	σ_β1β5_	=	0.010	0.003	0.003	−0.008	−0.007
	σ_β1β2_	σ^2^_β2_	σ_β2β3_	σ_β2β4_	σ_β2β5_		0.003	0.019	0.006	−0.019	−0.006
	σ_β1β3_	σ_β2β3_	σ^2^_β3_	σ_β3β4_	σ_β3β5_		0.003	0.006	0.054	−0.006	−0.054
	σ_β1β4_	σ_β2β4_	σ_β3β4_	σ^2^_β4_	σ_β4β5_		−0.008	−0.019	−0.006	0.036	0.009
	σ_β1β5_	σ_β2β5_	σ_β3β5_	σ_β4β5_	σ^2^_β5_		−0.007	−0.006	−0.054	0.009	0.104

Assuming a constant baseline hazard h_0_(t) which followed an exponential distribution, we used these randomly sampled coefficients in the above Cox regression to generate survival times (T_1i_, T_2i_, T_3i_) and classification variables (Y_1i_, Y_2i_, Y_3i_) for each simulated participant. Censoring due to non-cardiovascular death was also assumed to follow an exponential distribution. Power was assessed as the number of simulations where a significant treatment heterogeneity test was found, divided by the total number of simulations. For the first series of simulations, the treatment effect for MI and cardiac arrest were kept constant at a hazard ratio of 0.70 while varying the treatment hazard ratio on cardiovascular death from 0.70 to 2.0. Clear treatment homogeneity within the composite outcome occurs when all outcomes have the same hazard ratio, and heterogeneity is observed to greater degrees as the hazard ratio of one outcome increases. Each of multiple simulated datasets were then be analyzed to determine the chance of detecting statistically significant composite treatment heterogeneity or power, for a given single heterogeneous component. This process was repeated holding the treatment effect for cardiovascular death and MI the same, and varying this for cardiac arrest. Lastly, the treatment effect for cardiovascular death and cardiac arrest were kept constant while the treatment effect for MI was varied.

Data were simulated and analyzed in R for Unix version 2.11.1 [28]. This was calculated over 1500 iterations per condition. Based of interaction term standard errors (σ = 0.2 to 0.3) from POISE[14], 1500 iterations should allow us to estimate an interaction term within a level of accuracy of 0.01 to 0.02, using a two-tailed type I error rate of 0.05 [29]. Example R code for this simulation is included in appendix S1.

Finally we demonstrated the use of a composite outcome heterogeneity test by re-analyzing the POISE [14] data using a marginal time-to-event model [23], [24], [27]. The overall heterogeneity test compared the effect of peri-operative beta-blockers vs. placebo on cardiovascular death compared to myocardial infarction, and non-fatal cardiac arrest compared to myocardial infarction. Contrasts were fit comparing the effect of beta-blockers for among the three outcome types. Further to this, we summarized the degree of heterogeneity using an “I^2^ type” test, taking the difference of chi-square value for the composite treatment heterogeneity test from its degrees of freedom as a percentage of the chi-square value itself. This test is typically used to quantify the degree of heterogeneity across different studies in meta-analyses [30]. The test can be interpreted as the percentage of total variation due to true differences (i.e. not chance) in treatment effects across the components of the composite outcome.

## Results

**Figure 2:**
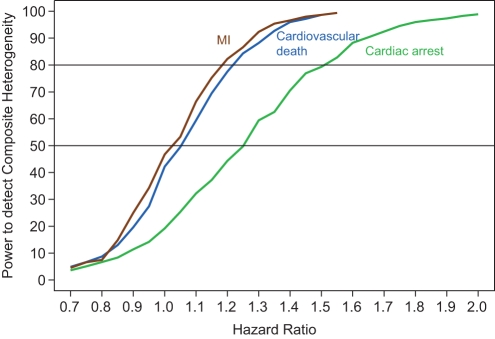
Power to detect treatment heterogeneity for each individual outcome within the composite outcome. Power to detect that the treatment hazard ratio for outcome is different from the remaining two outcomes, as it hazard ratio varied from 0.70 to 2.0 (horizontal axis). The hazard ratios for the other two outcomes are kept constant at 0.70. Each outcome is represented by a different power curve.

Figure 2 displays the power to detect treatment heterogeneity within the composite outcome as a function of the treatment effect for each outcome in the composite for our simulated trial. As expected, for all three outcomes the power to detect treatment heterogeneity within the composite outcome increased as a single outcomes' hazard ratio become more different from the remaining two. There was 50% power to detect that MI had a hazard ratio of 1.03 and 80% power to detect a hazard ratio of 1.18. There was 50% and 80% power to detect that cardiovascular death has larger hazard ratios of 1.06 and 1.22, respectively. Lastly, this simulated trial had the lowest power to detect that cardiac arrest had a different treatment effect compared to the other two outcomes, with 50% power to detect a hazard ratio of 1.25 and 80% power for a hazard ratio of 1.51.

Therefore, with this simulated study design there is some power to detect one outcome within the composite to be in the neutral to harmful range, depending on which outcome. This design would have little chance of demonstrating differences between the outcomes if all showed varying degrees of benefit due to treatment. The amount of power for composite treatment heterogeneity did depend on the standard error of the interaction term being manipulated, with power being greatest for a comparison of cardiovascular death versus MI (and reverse) as compared to cardiac arrest versus MI (since σ^2^_β4_<σ^2^_β5_).

For the actual POISE trial results [14] the interaction of treatment with outcome type was statistically significant, indicating composite outcome heterogeneity (p = 0.0072) (see table 2). Contrasts across the composite components provide evidence for a difference in treatment effect for cardiovascular death when compared to myocardial infarction (p = 0.0024), but no statistically significant difference for cardiac arrest compared to myocardial infarction, although there were relatively few cardiac arrests. For this effect, the value of I^2^ = 79.8 (95% CI: 36.3% to 93.6%), indicating a large amount of heterogeneity [30]. These results re-enforce the treatment pattern observed for the individual components in figure 1.

**Table 2:** Composite outcome treatment heterogeneity test results for the POISE trial .

Heterogeneity Test for Treatment Effect	p-value
Overall Composite	0.0072
Cardiovascular death vs. MI	0.0024
Cardiac arrest vs. MI	0.1976

Results of heterogeneity tests for the actual trial data.

## Discussion

Trialists may find this new test useful in planning and analyzing trials that use composite outcomes. At the design stage, trialists could explore the degree of treatment differences that could be detected for each outcome within the composite, given estimated outcome rates and covariances. Such power calculations are possible, even for complex composite heterogeneity patterns across multiple individual outcomes. This information may be considered in selecting the final trial design and sample size. If trial sample size cannot be altered based on this knowledge, then at least trialists can be informed of the degree of composite treatment heterogeneity they can detect with their current design. If a trial has very little power to detect a statistically different treatment effect for one outcome, for example non-fatal cardiac arrest in POISE[14], then this may inform and change our discussion of trial results.

A composite outcome heterogeneity test may be an additional piece of information that readers can consider when interpreting a trial results for a composite outcome. Such a test could help us distinguish real differences in outcome treatment effects within a composite from mere random variation. When readers examine treatment estimates on the individual outcomes in the composite, a heterogeneity test may discourage them from interpreting minor variations in treatment estimates as real differences between outcomes. Use of such a test itself may reinforce the play of chance on individual outcome results within a trial, as was done for subgroup analysis [11], [12], [31].

It would be beneficial to include discussion of possible treatment differences within a composite outcome in the trial pre-specified statistical analysis. Any comprehensive statistical analysis plan should define the assumptions of the models that will be used and suggest alternative models to be substituted if these assumptions are not met. As in any statistical analysis, the appropriate model assumptions must be examined prior to estimation of the treatment effect, to avoid a biased treatment estimate. For example, when using a linear regression the analyst must check for normality and independence of the error terms [32]. When using a proportional hazards model, the assumption of proportional hazards must be examined prior to model fitting [26]. Similarly, for a model analyzing a composite outcome, formed based on the assumption of homogeneity of treatment effect across its components, researchers would not want to emphasize the estimated treatment effect from the composite outcome if it were not a reasonable estimate of the overall effect. Guidance to distinguish random variation in treatment effects from important outcome differences may help in this decision. If there is evidence of composite heterogeneity, it may be unwise to proceed with the typically model. The composite outcome result could be presented along side with the treatment heterogeneity test result and possible I^2^ value, to clarify it interpretation. This may be followed by a discussion of evidence for and against the initial treatment homogeneity assumption. This observed effect may lead to further exploration of the mechanisms of action for the treatment being investigated. It could also guide the selection composite outcomes for future trials.

More research is needed to investigate tests of composite outcome treatment heterogeneity for a variety of outcome types and RCT designs. Our power calculations have assumed that the estimates of both outcome rates and the associations between survival times from a past trial accurately estimate these for future trials. One could also do sensitivity analyses to see how the power for this test would change if these were over-estimates or under-estimates. It would be helpful if published studies included information about the association or correlation between the components of commonly used composite outcomes, in addition to the composite outcome event rate itself. There is also merit to studying power to detect treatment differences between individual outcomes within a composite when this effect of treatment is not constant over time. Treatments that may show early benefit but later harm would likely require incorporating time into the heterogeneity test, forming outcome type by treatment by time interaction term, but further research would be needed to explore this scenario. Finally, we have applied the methods described to a single RCT. POISE [14] is only one example where a composite outcome heterogeneity test may have assisted in interpretation of trial results, and there may be other trials where such a test may be useful as well. This limits our inference and there is a need to apply these methods to more trials to provide greater insight about the patterns of treatment heterogeneity that commonly occurs in composite outcomes and the broader applicability of our proposed method.

Some may view the disadvantages of composite outcomes as outweighing their advantages. Our perspective is that although the disadvantages are real, composite outcomes will remain a reality for most RCTs. In fact, most outcomes that appear as single outcomes are composites of heterogeneous events. For example, the single primary outcome of stroke will usually be a composite of major and minor strokes or different types of stroke (e.g. intra-cerebral bleed, cerebral infarction, etc.) that occur at different frequencies and that may differ in their prognostic importance to patients. Even total mortality is a composite of different types of deaths, each of which may vary in response to a treatment. Despite the limitations of composite endpoints, the beneficial aspects related to sample size, cost, and clinical relevance make a persuasive argument for the continued use of composite outcomes in future trials. Therefore there is a need for guidance on how to determine when a composite outcome may not be appropriate to use and interpret for an individual RCT.

It is clear that a new direction is needed for the analysis of composite outcomes. The methods outlined in this manuscript provide a possible framework for approaching this problem and may help us to use and interpret composite outcomes more wisely.

## Supporting Information

Appendix S1 **R code to calculate power composite outcome heterogeneity test.** The following will calculate the power to detect composite outcome treatment heterogeneity if treatment does not change cardiovascular death (β4 = 0 or hazard ratio = 1.0) and both MI and non-fatal cardiac arrest have a treatment hazard ratio = 0.7 (interaction term β5 = 0): COpower(1500,4100,−0.35667,−1.43508,−2.35138,0.35667,0). The following will calculate the power to detect composite outcome treatment heterogeneity if treatment does not change non-fatal cardiac arrest (β5 = 0 or hazard ratio = 1.0) and both MI and cardiovascular death have a treatment hazard ratio = 0.7 (interaction term β4 = 0): COpower(1500,4100,−0.35667,−1.43508,−2.35138,0,0.35667). The following will calculate the power to detect composite outcome treatment heterogeneity if treatment does not change MI (β1 = 0 or hazard ratio = 1.0) and both non-fatal cardiac arrest and cardiovascular death have a treatment hazard ratio = 0.7 (interaction terms β4 = β5): COpower(1500,4100,0,−1.43508,−2.35138,−0.35667,−0.35667).
(DOC)
