# Supplementary material for: Designing and Analyzing Clinical Trials with Composite Outcomes: Consideration of Possible Treatment Differences between the Individual Outcomes
Source: PLoS One. 2012 Apr 17;7(4):e34785. doi: 10.1371/journal.pone.0034785 (PMC3328496; doi:10.1371/journal.pone.0034785)
Supplement: Appendix S1 — R code to calculate power composite outcome heterogeneity test. The following will calculate the power to detect composite outcome treatment heterogeneity if treatment does not change cardiovascular death (β4 = 0 or hazard ratio = 1.0) and both MI and non-fatal cardiac arrest have a treatment hazard ratio = 0.7 (interaction term β5 = 0): COpower(1500,4100,−0.35667,−1.43508,−2.35138,0.35667,0). The following will calculate the power to detect composite outcome treatment heterogeneity if treatment does not change non-fatal cardiac arrest (β5 = 0 or hazard ratio = 1.0) and both MI and cardiovascular death have a treatment hazard ratio = 0.7 (interaction term β4 = 0): COpower(1500,4100,−0.35667,−1.43508,−2.35138,0,0.35667). The following will calculate the power to detect composite outcome treatment heterogeneity if treatment does not change MI (β1 = 0 or hazard ratio = 1.0) and both non-fatal cardiac arrest and cardiovascular death have a treatment hazard ratio = 0.7 (interaction terms β4 = β5): COpower(1500,4100,0,−1.43508,−2.35138,−0.35667,−0.35667). (DOC) [file pone.0034785.s001.doc]

**Appendix S1**

library(survival)

library(mvtnorm)

COpower<-function(nrep,size,b1,b2,b3,b4,b5)

{

# initialize variables to record results

result<-c(0,0,0,0,0,0,0,0)

FalsePos <-0

COpower<-0

# Set MI outcome rate and rate of censoring due to Non-CV death

LambdaC1<- log(1-0.06) * -1

LambdaD<-log(1-0.01) * -1

# Estimates for β1 to β5 and the variance-covariance matrix Σ

mu<-c(b1,b2,b3,b4,b5)

cov<-matrix(

c(0.010, 0.003, 0.003,-0.008,-0.007,

0.003, 0.019, 0.006,-0.019,-0.006,

0.003, 0.006, 0.054,-0.006,-0.054,

-0.008,-0.019,-0.006, 0.036, 0.009,

-0.007,-0.006,-0.054, 0.009, 0.104),5,5,)

for(j in 1:nrep)

{

# Assume a multivariate normal distribution for β’s and create a random

# sample

beta<-rmvnorm(size,mu,cov)

beta1<-beta[,1]

beta2<-beta[,2]

beta3<-beta[,3]

beta4<-beta[,4]

beta5<-beta[,5]

d1<-c(rep(0,size))

d2<-c(rep(0,size))

d3<-c(rep(0,size))

surv1<-c(rep(0,size))

surv2<-c(rep(0,size))

surv3<-c(rep(0,size))

trt<-c(rep(0,size))

pid<-c(1:size)

for(i in 1:size)

{

# Assume ½ of patients in control group and ½ in treated group

trt[i]<-0

if(i>(size / 2)) trt[i]<-1

# Create survival times and indicator variables for each outcome

# using the randomly sampled β’s

L<-rweibull(1,shape=1,scale=LambdaD)

eventtime1<-rweibull(1,shape=1,scale=LambdaC1*

exp(-1 * (beta1[i] * trt[i])))

eventtime2<-rweibull(1,shape=1,scale=LambdaC1*

exp(-1 * (beta1[i] * trt[i] + beta2[i] + beta4[i]*trt[i])))

eventtime3<-rweibull(1,shape=1,scale=LambdaC1*

exp(-1 * (beta1[i] * trt[i] + beta3[i] + beta5[i]*trt[i])))

if(eventtime2<=L) L<-eventtime2

d1[i]<-0

if(eventtime1<=L) d1[i]<-1

surv1[i]<-min(eventtime1,L)

d2[i]<-0

if(eventtime2<=L) d2[i]<-1

surv2[i]<-min(eventtime2,L)

d3[i]<-0

if(eventtime3<=L) d3[i]<-1

surv3[i]<-min(eventtime3,L)

}

# Build indicator variables for regression

outcome0<-c(rep(0,size))

outcome1<-c(rep(1,size))

interact0<-outcome0*trt

interact1<-outcome1*trt

id<-c(pid,pid,pid)

treat<-c(trt,trt,trt)

censor<-c(d1,d2,d3)

time<-c(surv1,surv2,surv3)

outcomeD<-c(outcome0,outcome1,outcome0)

outcomeC<-c(outcome0,outcome0,outcome1)

interactD<-treat * outcomeD

interactC<-treat * outcomeC

# Perform marginal Cox regression

CEdata<-data.frame(id,treat,time,censor,outcomeD,outcomeC,

interactD,interactC)

fit2<-coxph(Surv(time,censor) ~treat + outcomeD + outcomeC + interactD + interactC + cluster(id),data=CEdata)

# Test for composite outcome heterogeneity

est<-matrix(c(fit2$coef[4:5]),1,2)

v<-fit2$var[4:5,4:5]

interact<-est%*%solve(v)%*%t(est)

p2<-(1-pchisq(interact,2))

if(p2<0.05) (FalsePos <- FalsePos + 1)

}

# Calculate Power for the heterogeneity test

COpower<- FalsePos / nrep

result<-c(b1,b2,b3,b4,b5,nrep,FalsePos,COpower)

write(result,file="result.txt",ncolumns=8, append=TRUE)

}
